# Optionality in Australian Football League draftee contracts

**DOI:** 10.1371/journal.pone.0291439

**Published:** 2023-09-14

**Authors:** Jemuel Chandrakumaran, Paul Larkin, Sam McIntosh, Sam Robertson

**Affiliations:** 1 Institute for Health and Sport (IHES), Victoria University, Melbourne, Australia; 2 Maribyrnong Sports Academy, Melbourne, Australia; 3 Western Bulldogs Football Club, Melbourne, Australia; Groupe ESC Dijon Bourgogne, FRANCE

## Abstract

Though player drafts have commonly been utilised to equitably disperse amateur talent and avoid bidding wars, often they have also been accused of creating a monopsony labour market which restricts player movement. Within the Australian Football League (AFL) some have called for the increase of the initial draftee contract from two to three seasons, which further pushes the envelope on monopsony power. Instead of increasing the contract length, this paper suggests a call option to be purchased by the teams allowing them to add a further season to the draftee contract at a predetermined compensation package should they choose to do so at the end of the initial contract. The call prices per pick were calculated using the Black-Scholes model and were valued between 1% and 1.5% of the pick value. However, it failed to follow a monotonic function similar to pick value, owing to managerial overconfidence and sunk investment plays. Overall, the findings allow teams to procure the option of increasing initial draftee contracts and not impede further on a player’s ability to move.

## Introduction

Pay and performance in the labour market has attracted the interest of many both in the solicitation of service and the employment/retention of talent. However, with most jobs requiring skills that ought to be developed, the cost of training has equally been brought into the debate. Though employers might like such costs to be borne by the apprentices exclusively, studies have suggested that the opposite materializes for a variety of reasons. Despite this, during the training period, employers do incur a net cost, as the wages paid to an apprentice would be higher than their marginal product, until a surplus is gained post proficiency (refer to Fig 1 in [1]). Hence, to guarantee recouping the investment in training, employers might resort to stricter terms, including but not limited to pre-employment contracts to extend post proficiency retention [2, 3].

However, within the professional services market, where employees have specific skills [4], employers unavoidably have to invest in training, as the failure to do so will result in unfavourable cost-benefit ratios (or the absence of a benefit [5]). As the personal services provided by such individuals are essentially unique to them, the market value for such talent could increase exponentially. Furthermore, given that labour market frictions such as wage compressions are more likely to happen with such employees, firms are endeavoured to invest in their upliftment [6]. Yet, how can an employer recoup their investment when they incubate such talent with an aim to elicit performance without impeding on the earnings potential of the employee? We aim to propose an alternative solution using the Australian Football League (AFL) player draft, where the proper investment in talent by teams could lead to strategic outcomes both on and off the field of play.

### The case of the AFL draft

In 1936, the National Football League (NFL) created the first player draft with the intent of creating an equitable mechanism for the allocation of amateur talent and avoid a bidding war for the athletes [7]. Teams take turns to select players in the reverse order of their regular season standing immediately prior to the draft, with the club finishing last receiving the first pick. After all teams make their respective selections (i.e., one round), the process is repeated again. The allocation of picks under this method is expected to cyclically alter the fortunes of each team within the league and increase the overall competitiveness of the sport [8]. Wanting to achieve the same, many sporting leagues across the world have incorporated versions of the player draft as competitive balance measures, including the AFL, who introduced this concept in 1986.

Irrespective of its benefits to the competition, the draft has been accused of inadvertently creating a monopsony market for amateur talent [9]. Amateurs signed on by clubs through the draft in most sporting leagues are usually restricted from moving to different teams within their initial contract period, which reduces their earnings potential in comparison to their veteran (or unrestricted) peers [10–15]. Clubs within Major League Baseball (MLB) use this provision to underpay these players, though teams have often cited training costs which indirectly increases their overall outgoings [16]. This has sparked multiple labour actions in the past including a successful legal challenge (*Adamson v New South Wales Rugby League Ltd (1991) 31 FCR 535*, *268)* in the Australian National Rugby League (NRL, the premier rugby league in Australia, with the second highest domestic sport viewership next to the AFL), where the court ruled the draft, (1) limited the freedom of a player to select their employer (Ibid, 280), and (2) imposed a new post-contractual restraint upon them (Ibid, 281). This begs the question on how the draft and subsequent player contracts could be augmented to suit the competing interests of the players, teams and the league overall.

Unlike most of its North American professional league counterparts, where initial draftee contracts are between three to four years, AFL teams provide a two-year contract for new draftees entering the league. However, with an increasing consensus that the initial two seasons does not allow teams to fully recoup their investments and retain amateur talent, clubs have lobbied for the contract length to be increased by one year [17], especially for players selected in the first round of the draft. The driving force behind this suggestion has been the earnings exponential secured by draftees from their third season, after the expiry of their initial contracts. Though the AFL draft has never been legally challenged, the proposed change would violate both clauses cited within the NRL precedent. Hence through this paper, we aim to introduce a model to value a team’s right to retain a draftee for the third season using financial option theory (similar to the NFL’s fifth year contract extension for draftees selected in the first round [18]). Essentially at the time of the draft, if a club wishes to retain a player for three seasons instead of two, they will purchase this right from the player using a call option, which they could exercise at the end of the initial draftee contract. This would then protect a team from engaging in a bidding war for the services of the player at the end of the two seasons and guarantee the player a fair compensation package for the third season. Hence the overarching aim of this paper is to, (1) identify the best alternative pick value method in the existing literature; (2) understand if it is in the best economic interest of a player to move to another club at any time during their career, which would warrant the need for higher compensation to retain the same; and (3) value the call option for each pick allowing teams to extend the initial draftee contract from two to three seasons.

### Pick value

Draft picks assigned to teams by the league each year are essentially financial instruments owned by the benefitting clubs. Teams can opt to either use the pick to select an amateur player or trade it for a player, another pick(s), or a selection(s) in the future. Given these picks are indivisible themselves and have varying levels of value associated to them, a litany of academic work has been presented in the past to value them. The first known draft pick value chart (PVC) was created by Mike McCoy and Jim Johnson of the Dallas Cowboys (an NFL club) in the late 1980s’ using a Weibull distribution model to equate draft day trades (i.e., where the earliest pick in each trade was equal to net of all other picks in the exchange). The proposed chart declined at an exponential rate where the difference between the first two picks was approximately 1/6^th^ of the first pick. However, as trades themselves include the biases of perceived value inferred upon them by decision makers (including overconfidence, confirmation bias etc.), the validity of the PVC has been questioned in the past [12].

Hence, Dawson and Magee [19] suggested that a pick value function ought to be both inverse and monotonic (a function that either never increases when inverse or decrease when direct), using an isotonic regression to predict the expected games played of draftees in the National Hockey League (NHL) based on the initial pick number used to select them. Their findings, while upholding their hypothesis, showed a lower rate of decline in pick value when compared to the PVC. Conlin and Emerson [20] expanded on this to use games started and active contract lengths as determinants of pick value in the NFL reconfirming the previous findings, similar to Schuckers who plotted this relationship with the help of local polynomial functions both in the NFL [21] and NHL [22]. Studies within the AFL have also used player ratings to determine pick value through linear regression models [23, 24] as the league’s own draft value index (DVI) mimicked certain properties in the NFL PVC. The common trend reciprocated by all of the aforementioned publications suggested that the indices used by the leagues over valued initial picks and that an ideal value function would decrease at a much lower rate.

### Data

For the purposes of this study, we used the survival estimate model proposed by Chandrakumaran [25] and Glasson and Bedford [26] as it enables the continuous evaluation of picks at different times in their careers. Survival models are commonly used in the pharmaceutical industry to evaluate the life expectancy of those involved in clinical trials. Similarly, we use this framework to determine the probability of a player to survive or continue in the league based on the pick used to select them. To replicate the methodology, the career metrics of all draftees selected between 2003 and 2016 and their retrospective performance from 2004 to 2017 was collected from Sorenson Technologies (a third-party data agency that provides data for the gambling industry sourced from the league). Although the draft has been in place since 1986, a decision was made to use post 2003 data owing to a variety of factors including, the completeness and accuracy of the data and the effectiveness of the draft itself. Prior to 2003, less than 80% of drafted players actually played at least one game in the league, and the number of recruits per season varied considerably. Moreover, with a majority of players selected after 2016 still playing in the league and the disruptions caused due to the pandemic, the subsection of newer recruits warranted to be excluded. Of the 1,123 draftees within this timeframe, players who previously played within the AFL (players who were delisted and have not secured a contract to play before the draft, are allowed to enter the draft in the AFL) and rookie elevations (teams use later picks to elevate players in their rookie lists to the senior squad, which might not reflect their actual worth), were removed from the sample to mitigate any biases, leaving a pool of 905 recruits. Based on the listing statics of players within this sample in Table 1, there is a clear drop in listed draftees from the third season. This could be due to decision makers delisting players who do not meet certain performance criterion after their initial contract phase. Moreover, the average pick (or selection) number drops each year as well, as teams only retain players who were selected early on in the draft, where the expected performance might be relatively high. It is also important to note that 48% of the sample was actively playing in the league as of 2018.

**Table 1:** Career listing outcomes of draftees and their respective performance since being drafted.

Years Since First Drafted	No. of Draftees Eligible to Play	No. of Draftees Listed in the year	No. of Draftees who Played in the year	Averages per year of listed draftees		
				Age	Pick	Games Played
1	905	905	531	19.17	33.69	4.91
2	833	828	610	20.14	33.3	7.73
3	766	686	575	21.12	31.48	9.84
4	697	570	504	22.09	30.03	11.77
5	638	447	408	23.05	28.34	12.59
6	578	371	335	24.03	28.06	13.18
7	513	289	267	24.99	26.83	13.74
8	445	226	213	25.94	26.09	14.06
9	382	172	160	26.92	25.08	14.65
10	312	126	121	27.92	25.32	14.53
11	249	87	80	28.85	26.38	13.97
12	179	45	39	29.62	21.69	13.16
13	118	23	21	30.57	21.91	12.39
14	57	6	6	31.33	33.67	10.83

### Design & results

The survival function, *S(t)*, for a player’s career having a length *T* of more than *t* games is given in Eq 1, where *F(t)* is the cumulative density function for that player. This timeframe is assumed to be the time in which a draftee is listed to play a game until they eventually retire or are delisted. The hazard event is excluded (or considered still active) if a player is delisted in one season and picked up by another team in a few years.


$$S(t)=P(T>t)=\int_{t}^{\infty }f(u)du=1-F(t)$$ (1)

**Fig 1:**
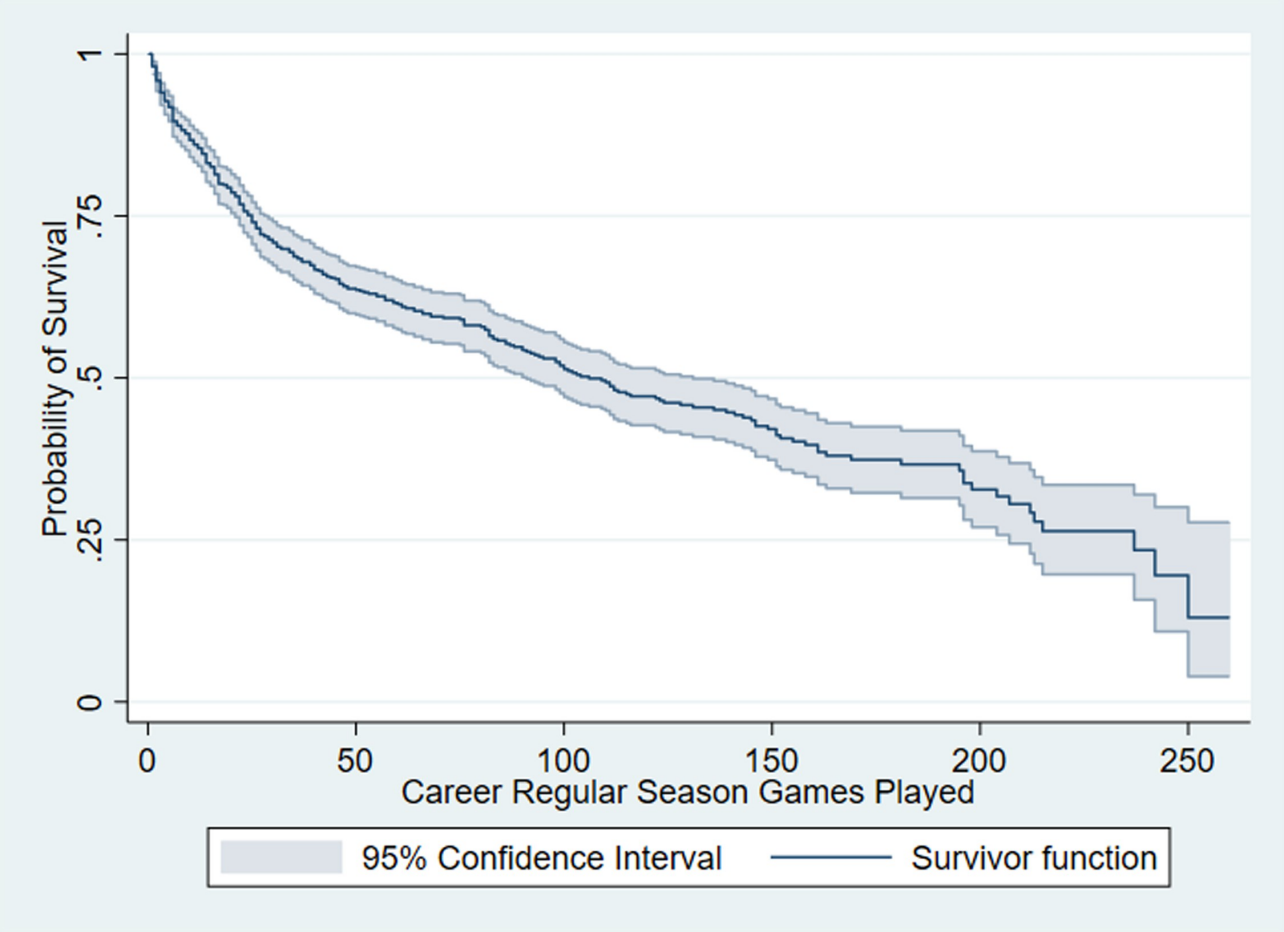
Kaplan-Meier survival estimate of draftees selected between 2003 and 2016.

The Kaplan-Meier survival estimate function of all draftees within the sample is given in Fig 1. This shows the probability of a draftee surviving in the league, should they play *t* regular season games. Based on the curve we can expect a draftee to play a median of just over 100 games in their career (at y = 0.5 refer to the value of x).

Whilst this allows the prediction of a draftee’s expectancy to survive based on the games they have played; a Cox proportional hazards model was employed to value each individual selection. Though a classical model always uses hazard rates we retrospectively augmented this to evaluate survival instead of hazard to keep in line with the theme, through Eq 2. Here *S*_*i*_*(0)* refers to player *i*’s probability to survive in the league, based on the estimated coefficient *(β*_*1*_*)* for the pick used to select them *(Pick*_*i*_*)*. Also, *S(0)* refers to the survival estimate of a draftee after playing zero games as observed in the Kaplan-Meier survival function given in Fig 1.


$$S_{i}(0)=1-\left((1-S(0))\;*\;\exp\left(\beta_{1}{\;\text{Pick}\;}_{i}\right)\right)$$ (2)

After setting the cross sectional data set as a survival panel, *S(0)* was estimated at 0.9808 with *β*_*1*_ retaining a value of 0.0274 (refer to the estimation results in S1 Table model (1) in S1 File). The calculated survival estimate per pick is given in S2 Table within the S1 File (column: Spot Price). This also mimicked the value function estimated in the previous studies [25, 26]. As a robustness check the Cox-Snell residuals were mapped in S1 Fig in S1 File, showing a Nelson-Aalen cumulative hazard following the prior closely at 45^0^ except at very large volumes. With a mean of 104 and a tail spanning across till 260, this variation in the larger volumes is deemed acceptable for the purposes of this study. Overall, the pick value function derived through this exercise upheld the inverse monotonic assumption laid out in the existing literature and will be used as the baseline model for the forthcoming sections.

### Transferring teams

**Fig 2:**
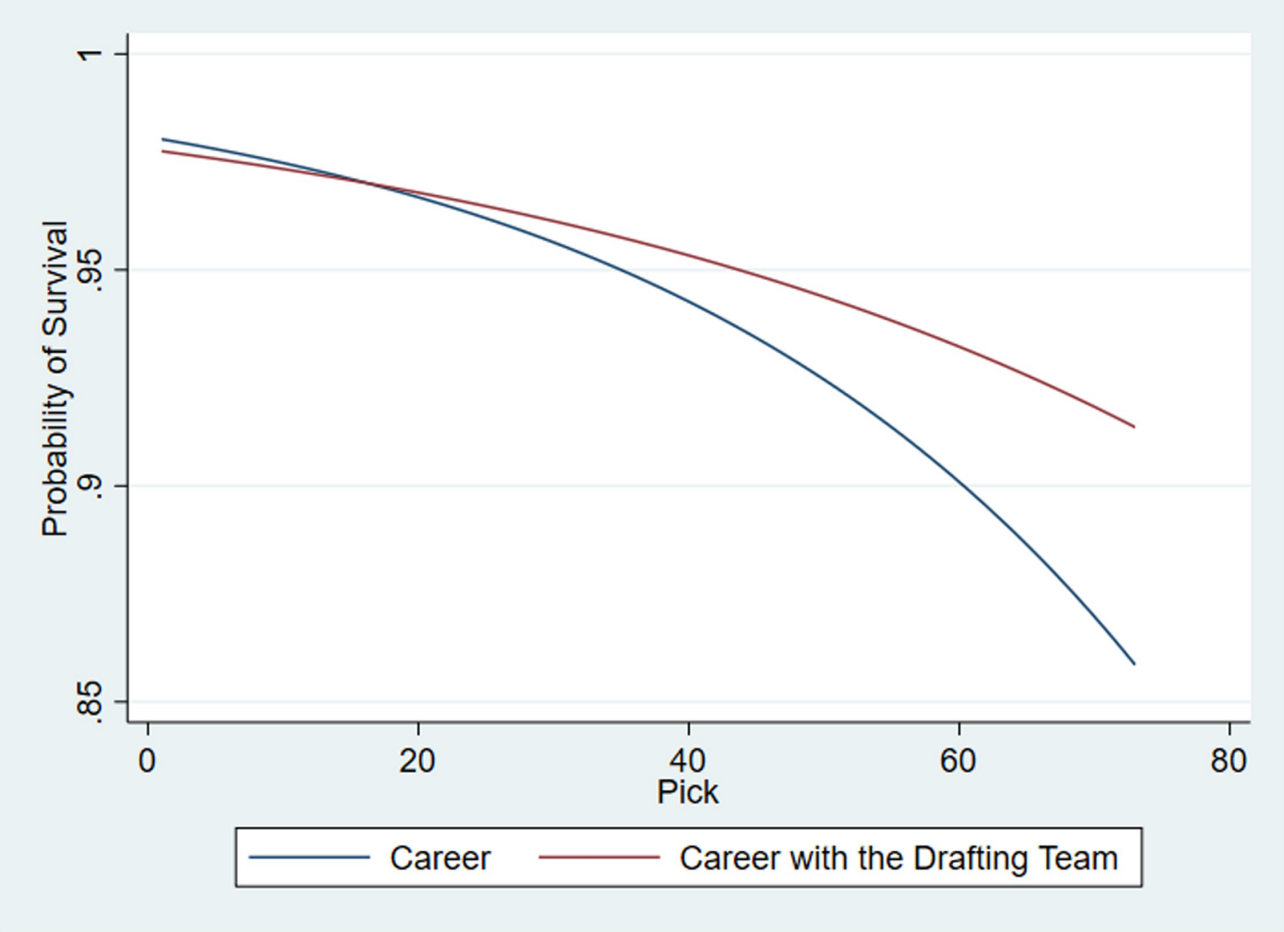
Survival estimates per draft selection at t = 0 in both their drafting team career and their entire career.

Prior to creating an option pricing model to incorporate the option to extend a contract to a third season, it is essential to understand if the option to explore moving teams is in the best economic interest of a player. On the other hand, players might also entertain the prospect of moving, to get the exposure of playing with a championship team, be listed in a lower rated team to get more consistent game time and even relocate closer to their families. Previous research within the AFL has suggested players who remain with the team that initially drafted them tend to provide 27 points per season to the team’s net point difference [27]. Yet, to verify this against each individual selection, the survival estimate model (Eq 2) used in the previous section was repeated utilizing the career regular season games of a recruit with the team that drafted them as per model (2) in S1 Table of S1 File and graphically denoted in Fig 2 together with the initial survival estimates for the whole career from the previous section (the robustness check which yielded a similar outcome is presented in S2 Fig in S1 File).

Overall, based on the curvature of the graphs, most players did perform better with the team that initially drafted them as demonstrated by the higher survival estimates. However, those who were drafted early on using picks one through to sixteen, which is the same group targeted by the proposed change, did have a slight advantage had they moved to a different team at any point in their career. Based on the collective bargaining agreement (CBA) for the period ending 2022 [28], this would also bring in increased economic returns for the player as well, as more games yielded more variable pay (however it is important to note that athletic pay in the sport industry is determined by considering various variables that may not predominantly relate to marginal productivity [29]). On the other hand, post pick sixteen, moving clubs proved to be detrimental for the player. This could be attributed to a variety of factors including, clubs providing lengthier lucrative contracts for early picks to leave their drafting clubs, managers or coaches giving early selections more game time to justify their investments, and teams having to drop players giving due consideration to roster and salary cap limits, whilst facilitating incoming transfers. The findings here highlight the need for players to be fairly compensated by teams in their contracts and eventual negotiations, especially if there are any restrictions on player movement.

### Option value

One suggestion that has been presented is to allow teams to retain a player they recruit through the draft for one more season after the end of the initial contract at season two. The proposal would eventuate between the team and the player at the time of the draft (*t = 0*). Hence, the best way to cater to this premature contract is through an option.

### Empirical framework

In theory, a call option would be purchased by the team selecting the player, agreeing to a pay them a predetermined salary for their third season at *t = 0*. This would inherently (1) protect the team from exponential pay increases observed amongst draftees at the end of season two (*t = 2*); whilst (2) allowing them to negate extending to a third season if they chose not to proceed with the player after their initial contract; and (3) give players the ability to negotiate a competitive compensation package for their third season subject to meeting a set of prerequisites. To explore this, the Black-Scholes model (BSM) was chosen [30] to value the call as it is quite commonly used with similar styled options (where the underlying asset will have a log normal distribution of prices following a random walk with a constant risk-free rate and volatility yielding no dividends during the life of the potion), the formula for which is given in Eq 3.

$$C=S_{t}N\left(d_{1t}\right)-Ke^{-rt}N\left(d_{2t}\right)$$ (3)

where

C = call option price

S_t_ = spot price

K = strike price

r = risk-free interest rate

t = time to maturity

σ_v_ = volatility

N = normal distribution

$$d_{1t}=\frac{\ln\frac{S_{t}}{K}+\left(r+\frac{\sigma_{v}^{2}}{2}\right)t}{\sigma_{s}\sqrt{t}}$$ (4)

$$d_{2t}=d_{1t}-\sigma_{s}\sqrt{t}$$ (5)

The BSM equation requires five key inputs. First of which is the spot price at *t = 0*. As this has already been calculated in the previous pick value section (i.e., model (1) from S1 Table in S1 File), the same method was reused to define its strike price at *t = 2*, which is the second input, utilizing the coefficients defined in S1 Table model (3) in S1 File (the robustness check which yielded a similar outcome is presented in S3 Fig in S1 File). Thirdly, the volatility of each selection was computed using the pre-defined spot and strike prices before moving onto reaffirming the time to maturity as two, which is the current length of the draftee contracts. In terms of the risk-free interest rate, which is the last of the five inputs, the average improvement in regular season win percentage was used. As the data used in this study utilizes performance metrics from 2004 to 2017 for draftees from 2003 to 2016, the average net improvement (*NI*) per year from the 2006 season was calculated as it will be the third season of the first draftees from 2003 (as Gold Coast and Greater Western Sydney entered the league after 2006, their first two seasons were excluded from the sample).

**Fig 3:**
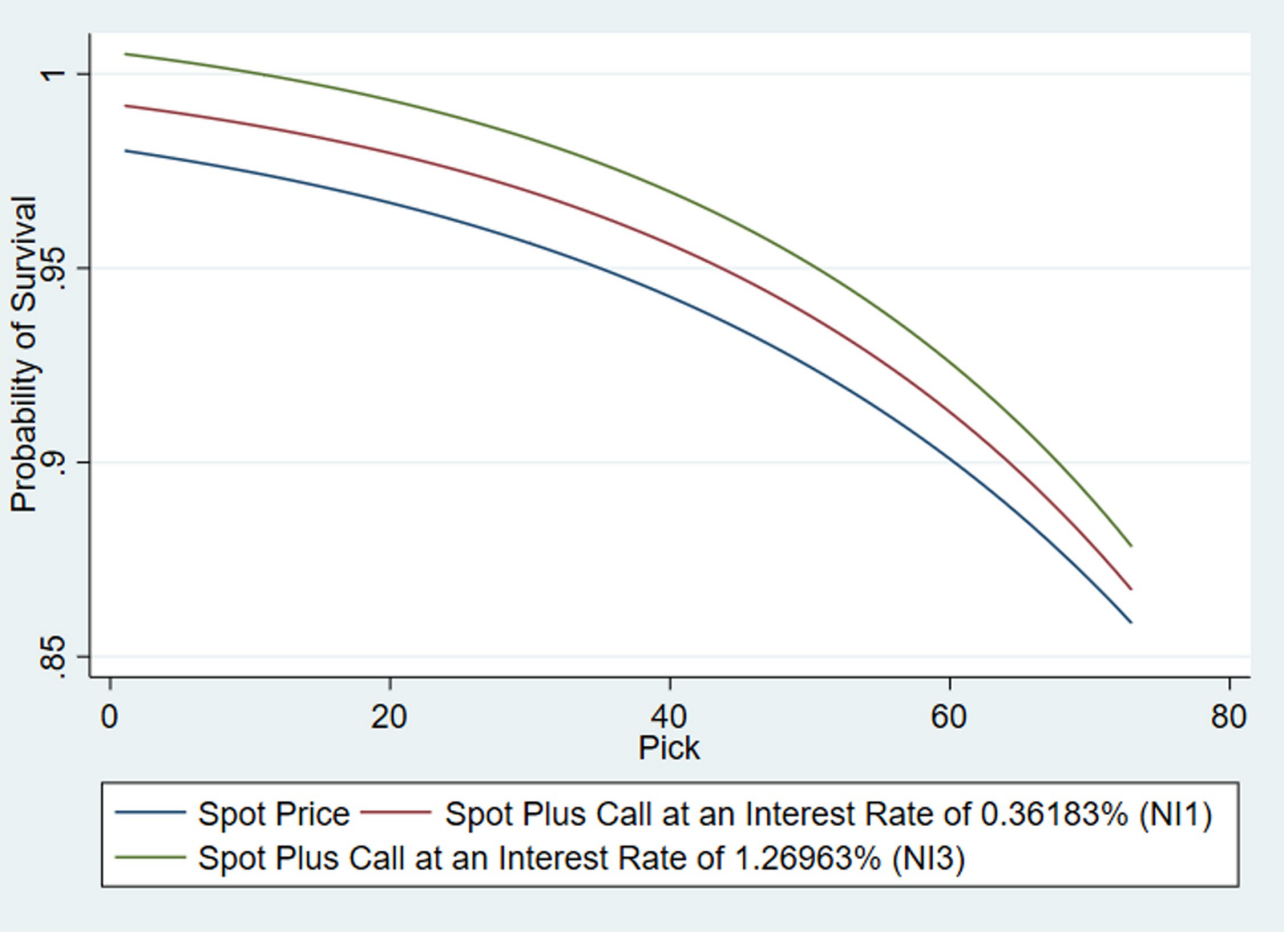
Career survival function (spot) & call option price.

The option prices based on the spot and strike were thus calculated and depicted in Fig 3 using two risk-free rates. The first being the average year-on-year increase in regular season winning percentage (*NI1*) and the second, the cumulative improvement over three seasons (*NI3*). Since the option considered here looks at the benefit to a team in extending the draftee contract by one year, the year-on-year risk-free rate (*NI1*) suited the purpose of the study.

The analysis shows both the spot and call price functions run parallel to each other. The calls themselves were valued between 1% and 1.5% of the spot price. However, as shown in S2 Table in S1 File, individual call prices increase from the first pick through to forty-seven before declining again. Ideally as career earnings are usually higher amongst players recruited early in the draft [31], the call price should be higher to represent the greater changes in compensation and then decline monotonically.

## Discussion

The question now remains as to why the option costs of these picks do not decline monotonically as the coinciding pick values. Research in psychology has suggested people are generally confident in their decisions [32]. This is especially exacerbated when they have access to more information, similar to talent assessors who evaluate amateurs using a variety of metrics, including scout reports, game performance, player interviews and draft combine performance [33]. Secondly, within financial theory Kahneman and Tversky [34] observed a phenomenon where people invest further resources to support a prior course of action when alternative options were available. Staw and Hoang [35] repurposed this in the National Basketball Association (NBA) stating that general managers tend to play draftees picked early on in the draft even when their on-court performance did not warrant the decision and coined it as sunk investment plays.

With these two human irrationalities driving the decision makers in the team, players drafted early on are likely to get more game time in the third season irrespective of their outcomes. This will reduce the deviation in game time observed amongst such players, which is one of the inputs of the BSM. On the other hand, players drafted in the middle would not be similarly privileged as teams would delist them (or play them less frequently) because there is a high variability in their output when compared to their initial selection and their initial financial commitment does not warrant riding the loss. Hence the higher volatility pushes the price of the call up [36, 37].

Still, this experiment introduced an alternative solution by which teams could extend draftee contracts without adding any additional contractual constraints on the player. To put this into practice, let’s assume that there is a team that wishes to purchase an option to extend the draftee contract of a player selected at pick one. If we assume that the player will be paid 0.9803 (refer to S1 Table in S1 File) in their entire lifetime, and 0.9753 of that would be paid from their third season, the team will have to pay 0.0116 (or 1.18%) of their expected total career earnings early on to purchase this option. At the end of the second season, the team can decide to exercise this option and force the player to play under the previously agreed terms or release them. Either way, the player has not been undercut of their potential and the team mitigates any upsides as well.

## Conclusion

In the professional sport market, teams are continuously faced with the issue of restocking their rosters. As veteran players enter the free agency market to increase their net worth, teams themselves dip into the same market, whilst also investing in long term amateur athlete development plans. Given the AFL holds the draft to disperse this non-professional talent equitably, clubs are permitted to restrict the players they draft for two seasons. However, with increasing calls for the option of extending this period from two to three years, it is imperative this is done in a way that does not financially disadvantage the players.

In a perfect world, amateur players usually perform better overall when they continue to play for the team that initially drafted them [23]. Yet, the key findings within this paper, while agreeing with this presupposition overall, suggested players selected through the first sixteen picks do tend to play more games (as represented by the slightly higher survival estimates) if they moved teams at any point in their careers. This would in turn increase the variable economic returns for the player. Hence it is plausible to assume that giving teams the ability to extend the initial contract period, even for one additional year, does indeed impose a ‘restraint of trade’ on the player.

In order to circumvent this, the optimal solution would be to give the teams the option to extend to the third season by purchasing this right at the time of the initial contract. Using the BSM, call option prices for each pick and their value as a percentage of the spot price were given in S2 Table in S1 File. This novel approach provides (1) players an additional inflow on top of the value of their pick (which is the price of the call); (2) teams the opportunity to negotiate third season wages today (*t = 0*) rather than at the end of season two (*t = 2*); and (3) clubs the option to withdraw from extending to third season if the draftee did not perform. Moreover, keeping these contracts and option to extend to relatively lower number of periods will discourage contract fatigue, and ensure draftees continuously deliver outcomes to match expectations [38]. On the other hand, allowing teams to take options on future services would guarantee higher compensation for the players in the long run, than when they initiate the options themselves [39].

Whilst previously out of reach, the growing incorporation of technical staff within sporting teams would allow for the simultaneous refreshing of this method and retrofit it to other time frames. These could be three-year contracts with an option to extend for another one or more years and also repurposed to any league as long as the expected outcomes increases every season. If the expected outcome in the next season is less than the previous one, teams are better off paying the effective rate for the player as there would be no need to hedge against pay increases. Similarly, before such extension options are proposed, it is important to evaluate if a player’s prospects in terms of playing time increases should they move to a different team. If not, a player would be better off playing for their drafting team as their prospects remain high. On the other hand, drafting teams could exert a higher level of monosponic exploitation as the marketability of their skill is low elsewhere.

Likewise, in the general labour market, firms requiring workers with specific skills would ideally invest in the required training and upliftment of apprentices. This would translate to a period where these firms will incur a net cost as the productivity of these apprentices, will not match their wages. However, upon achieving said proficiency, the firm will make a profit through the surplus (difference between apprentice wages and marginal productivity). Still, should the apprentice depart the firm upon completing the training (to join a rival firm), the firm will break even on their total investment, or even make a net loss (refer to Fig 1 in [1]). This will lead the firm to be in a constant state of training without sustaining a proficient cohort in its workforce [40]. Through this paper, we have suggested a way by which organisations could recoup on the surplus, using post traineeship workers for a smaller period of time, maintaining a balance between skilled workers and apprentices.

## Supporting information

S1 File (DOCX)

S1 Data (XLSX)
