# Supplementary material for: Optionality in Australian Football League draftee contracts
Source: PLoS One. 2023 Sep 14;18(9):e0291439. doi: 10.1371/journal.pone.0291439 (PMC10501648; doi:10.1371/journal.pone.0291439)
Supplement: S1 File — (DOCX) [file pone.0291439.s001.docx]

# **Supporting Information**

**S1 Fig: Fitted Residuals of the Career Games (t=0) Estimation**


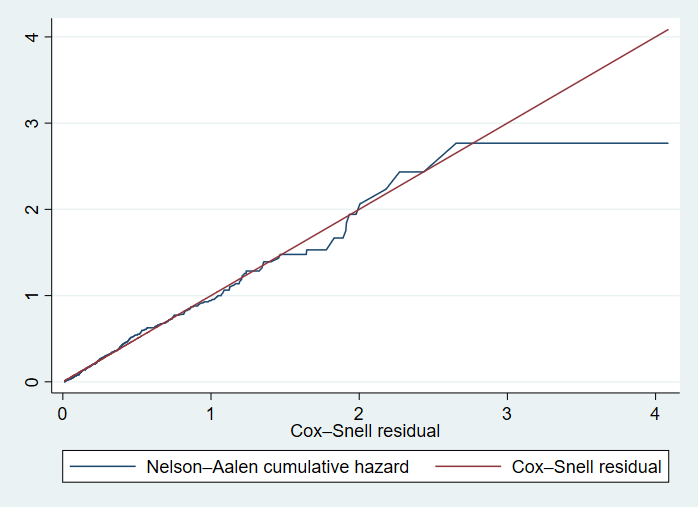


**S2 Fig: Fitted Residuals of the Drafting Team Games (t=0) Estimation**


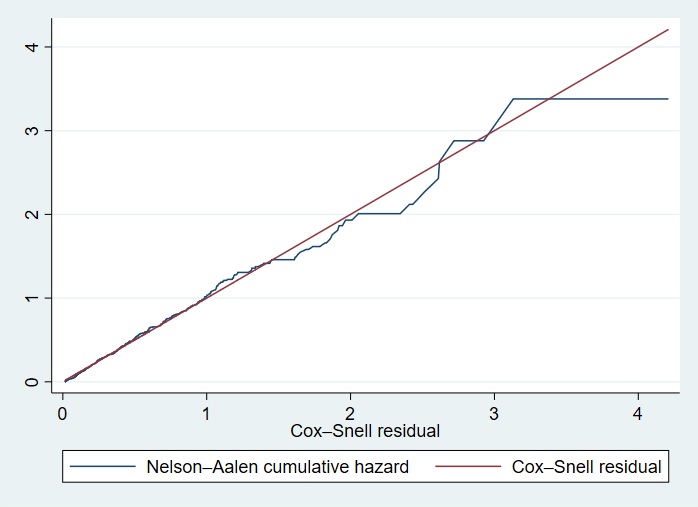


**S3 Fig: Fitted Residuals of the Career Games (t=2) Estimation**


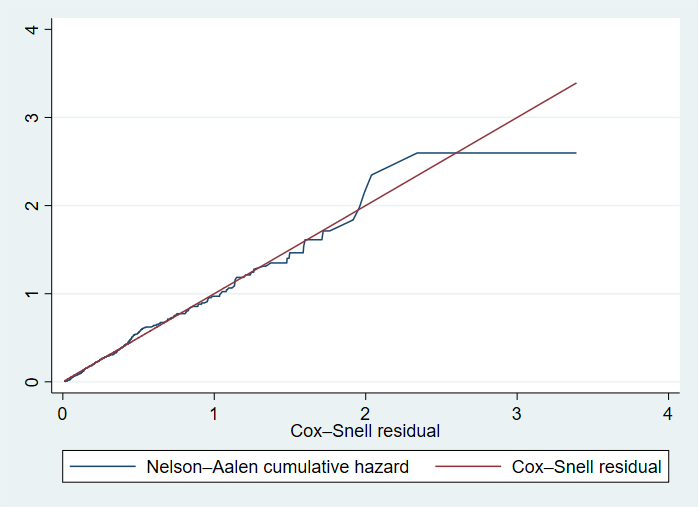


**S1 Table: Career Survival Models and Estimated S(0) and β_1_ values**

| S(0) | Scenario | Model | Hazard Ratio for Pick (β_1_) | Std. Err. | P \|z\| | No. of Obs. |
| --- | --- | --- | --- | --- | --- | --- |
| 0.9808 | Career Games | 1 | 1.0274 | 0.0028 | 0.0000 | 782 |
| 0.9779 | Career Games with Drafting Team | 2 | 1.0187 | 0.0024 | 0.0000 | 770 |
| 0.9759 | Career Games after Season 2 | 3 | 1.0245 | 0.0031 | 0.0000 | 622 |

**S2 Table: Spot and Option Prices**

| Pick | Spot Price | Strike Price |  | Call - Put Option Price to Extend with a Risk-Free Rate of | |  | % of Spot Price | |
| --- | --- | --- | --- | --- | --- | --- | --- | --- |
|  |  |  |  | 0.36183% (NI1) | 1.26963% (NI3) |  | NI1 | NI3 |
| 1 | 0.9803 | 0.9753 |  | 0.0116 | 0.0249 |  | 1.1810% | 2.5389% |
| 2 | 0.9797 | 0.9747 |  | 0.0116 | 0.0250 |  | 1.1888% | 2.5494% |
| 3 | 0.9792 | 0.9741 |  | 0.0117 | 0.0251 |  | 1.1966% | 2.5599% |
| 4 | 0.9786 | 0.9734 |  | 0.0118 | 0.0252 |  | 1.2045% | 2.5704% |
| 5 | 0.9780 | 0.9728 |  | 0.0119 | 0.0252 |  | 1.2123% | 2.5808% |
| 6 | 0.9774 | 0.9721 |  | 0.0119 | 0.0253 |  | 1.2201% | 2.5913% |
| 7 | 0.9767 | 0.9714 |  | 0.0120 | 0.0254 |  | 1.2279% | 2.6017% |
| 8 | 0.9761 | 0.9707 |  | 0.0121 | 0.0255 |  | 1.2357% | 2.6120% |
| 9 | 0.9754 | 0.9699 |  | 0.0121 | 0.0256 |  | 1.2434% | 2.6224% |
| 10 | 0.9748 | 0.9692 |  | 0.0122 | 0.0257 |  | 1.2512% | 2.6326% |
| 11 | 0.9741 | 0.9684 |  | 0.0123 | 0.0257 |  | 1.2589% | 2.6429% |
| 12 | 0.9733 | 0.9677 |  | 0.0123 | 0.0258 |  | 1.2665% | 2.6530% |
| 13 | 0.9726 | 0.9669 |  | 0.0124 | 0.0259 |  | 1.2742% | 2.6631% |
| 14 | 0.9718 | 0.9660 |  | 0.0125 | 0.0260 |  | 1.2817% | 2.6731% |
| 15 | 0.9711 | 0.9652 |  | 0.0125 | 0.0261 |  | 1.2892% | 2.6831% |
| 16 | 0.9703 | 0.9643 |  | 0.0126 | 0.0261 |  | 1.2967% | 2.6929% |
| 17 | 0.9694 | 0.9634 |  | 0.0126 | 0.0262 |  | 1.3040% | 2.7026% |
| 18 | 0.9686 | 0.9625 |  | 0.0127 | 0.0263 |  | 1.3113% | 2.7122% |
| 19 | 0.9677 | 0.9616 |  | 0.0128 | 0.0263 |  | 1.3185% | 2.7217% |
| 20 | 0.9668 | 0.9606 |  | 0.0128 | 0.0264 |  | 1.3256% | 2.7310% |
| 21 | 0.9659 | 0.9597 |  | 0.0129 | 0.0265 |  | 1.3326% | 2.7402% |
| 22 | 0.9650 | 0.9587 |  | 0.0129 | 0.0265 |  | 1.3395% | 2.7492% |
| 23 | 0.9640 | 0.9576 |  | 0.0130 | 0.0266 |  | 1.3462% | 2.7581% |
| 24 | 0.9630 | 0.9566 |  | 0.0130 | 0.0266 |  | 1.3528% | 2.7668% |
| 25 | 0.9620 | 0.9555 |  | 0.0131 | 0.0267 |  | 1.3592% | 2.7753% |
| 26 | 0.9609 | 0.9544 |  | 0.0131 | 0.0267 |  | 1.3655% | 2.7835% |
| 27 | 0.9598 | 0.9533 |  | 0.0132 | 0.0268 |  | 1.3716% | 2.7915% |
| 28 | 0.9587 | 0.9521 |  | 0.0132 | 0.0268 |  | 1.3776% | 2.7993% |
| 29 | 0.9576 | 0.9509 |  | 0.0132 | 0.0269 |  | 1.3833% | 2.8069% |
| 30 | 0.9564 | 0.9497 |  | 0.0133 | 0.0269 |  | 1.3888% | 2.8141% |
| 31 | 0.9552 | 0.9485 |  | 0.0133 | 0.0269 |  | 1.3941% | 2.8210% |
| 32 | 0.9539 | 0.9472 |  | 0.0133 | 0.0270 |  | 1.3991% | 2.8277% |
| 33 | 0.9526 | 0.9459 |  | 0.0134 | 0.0270 |  | 1.4039% | 2.8340% |
| 34 | 0.9513 | 0.9445 |  | 0.0134 | 0.0270 |  | 1.4083% | 2.8399% |
| 35 | 0.9500 | 0.9431 |  | 0.0134 | 0.0270 |  | 1.4125% | 2.8455% |
| 36 | 0.9486 | 0.9417 |  | 0.0134 | 0.0270 |  | 1.4164% | 2.8506% |
| 37 | 0.9472 | 0.9403 |  | 0.0134 | 0.0270 |  | 1.4199% | 2.8553% |
| 38 | 0.9457 | 0.9388 |  | 0.0135 | 0.0270 |  | 1.4230% | 2.8596% |
| 39 | 0.9442 | 0.9373 |  | 0.0135 | 0.0270 |  | 1.4258% | 2.8634% |
| 40 | 0.9427 | 0.9357 |  | 0.0135 | 0.0270 |  | 1.4282% | 2.8666% |
| 41 | 0.9411 | 0.9341 |  | 0.0135 | 0.0270 |  | 1.4301% | 2.8693% |
| 42 | 0.9394 | 0.9325 |  | 0.0134 | 0.0270 |  | 1.4316% | 2.8715% |
| 43 | 0.9377 | 0.9308 |  | 0.0134 | 0.0269 |  | 1.4325% | 2.8730% |
| 44 | 0.9360 | 0.9291 |  | 0.0134 | 0.0269 |  | 1.4330% | 2.8739% |
| 45 | 0.9342 | 0.9273 |  | 0.0134 | 0.0269 |  | 1.4329% | 2.8741% |
| 46 | 0.9324 | 0.9255 |  | 0.0134 | 0.0268 |  | 1.4323% | 2.8736% |
| 47 | 0.9305 | 0.9237 |  | 0.0133 | 0.0267 |  | 1.4310% | 2.8723% |
| 48 | 0.9286 | 0.9218 |  | 0.0133 | 0.0267 |  | 1.4290% | 2.8702% |
| 49 | 0.9266 | 0.9199 |  | 0.0132 | 0.0266 |  | 1.4264% | 2.8672% |
| 50 | 0.9246 | 0.9179 |  | 0.0132 | 0.0265 |  | 1.4231% | 2.8633% |
| 51 | 0.9225 | 0.9158 |  | 0.0131 | 0.0264 |  | 1.4190% | 2.8584% |
| 52 | 0.9204 | 0.9137 |  | 0.0130 | 0.0263 |  | 1.4141% | 2.8525% |
| 53 | 0.9182 | 0.9116 |  | 0.0129 | 0.0261 |  | 1.4083% | 2.8455% |
| 54 | 0.9159 | 0.9094 |  | 0.0128 | 0.0260 |  | 1.4016% | 2.8373% |
| 55 | 0.9136 | 0.9072 |  | 0.0127 | 0.0258 |  | 1.3939% | 2.8279% |
| 56 | 0.9112 | 0.9049 |  | 0.0126 | 0.0257 |  | 1.3852% | 2.8171% |
| 57 | 0.9087 | 0.9025 |  | 0.0125 | 0.0255 |  | 1.3754% | 2.8050% |
| 58 | 0.9062 | 0.9001 |  | 0.0124 | 0.0253 |  | 1.3645% | 2.7913% |
| 59 | 0.9036 | 0.8976 |  | 0.0122 | 0.0251 |  | 1.3524% | 2.7761% |
| 60 | 0.9009 | 0.8950 |  | 0.0121 | 0.0249 |  | 1.3390% | 2.7592% |
| 61 | 0.8981 | 0.8924 |  | 0.0119 | 0.0246 |  | 1.3242% | 2.7404% |
| 62 | 0.8953 | 0.8898 |  | 0.0117 | 0.0243 |  | 1.3079% | 2.7196% |
| 63 | 0.8924 | 0.8870 |  | 0.0115 | 0.0241 |  | 1.2901% | 2.6968% |
| 64 | 0.8894 | 0.8842 |  | 0.0113 | 0.0238 |  | 1.2707% | 2.6716% |
| 65 | 0.8864 | 0.8814 |  | 0.0111 | 0.0234 |  | 1.2495% | 2.6441% |
| 66 | 0.8832 | 0.8784 |  | 0.0108 | 0.0231 |  | 1.2263% | 2.6138% |
| 67 | 0.8800 | 0.8754 |  | 0.0106 | 0.0227 |  | 1.2012% | 2.5806% |
| 68 | 0.8766 | 0.8723 |  | 0.0103 | 0.0223 |  | 1.1738% | 2.5443% |
| 69 | 0.8732 | 0.8691 |  | 0.0100 | 0.0219 |  | 1.1441% | 2.5045% |
| 70 | 0.8697 | 0.8659 |  | 0.0097 | 0.0214 |  | 1.1117% | 2.4608% |
| 71 | 0.8661 | 0.8625 |  | 0.0093 | 0.0209 |  | 1.0765% | 2.4128% |
| 72 | 0.8624 | 0.8591 |  | 0.0090 | 0.0204 |  | 1.0381% | 2.3599% |
| 73 | 0.8586 | 0.8556 |  | 0.0086 | 0.0198 |  | 0.9960% | 2.3014% |
